# Pirfenidone and nintedanib modulate properties of fibroblasts and myofibroblasts in idiopathic pulmonary fibrosis

**DOI:** 10.1186/s12931-016-0328-5

**Published:** 2016-02-04

**Authors:** Siri T. Lehtonen, Anniina Veijola, Henna Karvonen, Elisa Lappi-Blanco, Raija Sormunen, Saara Korpela, Ulrika Zagai, Magnus C. Sköld, Riitta Kaarteenaho

**Affiliations:** Department of Anatomy and Cell Biology, Cancer and Translational Medicine Research Unit, University of Oulu, Aapistie 7 A, FIN-90 220 Oulu, Finland; Department of Internal Medicine, Respiratory Research Unit and Medical Research Center, Oulu University Hospital, Aapistie 5 A, FIN-90220 Oulu, Finland; Laboratory of Tissue Repair and Regeneration, Matrix Dynamics Group, Faculty of Dentistry, University of Toronto, 150 College Street, Toronto, ON M5S 3E2 Canada; Department of Pathology, Oulu University Hospital, P.O. Box 50, FIN90029 Oulu, Finland; Department of Pathology, Cancer and Translational Medicine Research Unit, University of Oulu, Aapistie 5 B, FIN-90220 Oulu, Finland; Biocenter Oulu, University of Oulu, Aapistie 5 A, FIN-90220 Oulu, Finland; Department of Medical Epidemiology & Biostatistics, Karolinska Institutet, SE-17177 Stockholm, Sweden; Respiratory Medicine Unit, Department of Medicine Solna and Centre for Molecular Medicine, Karolinska Institutet, SE-17177 Stockholm, Sweden; Research Unit of Internal Medicine, Respiratory Research Unit and Medical Research Center, University of Oulu, Aapistie 5 A, FIN-90220 Oulu, Finland; Unit of Medicine and Clinical Research, Pulmonary Division, University of Eastern Finland, Kuopio, Finland; Center for Medicine and Clinical Research, Division of Respiratory Medicine, Kuopio University Hospital, Kuopio, Finland

**Keywords:** Cell culture, Ultrastructure, Usual interstitial pneumonia, UIP

## Abstract

**Background:**

Idiopathic pulmonary fibrosis (IPF) is an incurable lung disease with a poor prognosis. Fibroblasts and myofibroblasts are the key cells in the fibrotic process. Recently two drugs, pirfenidone and nintedanib, were approved for clinical use as they are able to slow down the disease progression. The mechanisms by which these two drugs act in in vitro cell systems are not known. The aim of this study was therefore to examine the effects of pirfenidone and nintedanib on fibroblasts and myofibroblasts structure and function established from patients with or without IPF.

**Methods:**

Stromal cells were collected and cultured from control lung (*n* = 4) or IPF (*n* = 7). The cells were treated with pirfenidone and/or nintedanib and the effect of treatment was evaluated by measuring cell proliferation, alpha smooth muscle actin (α-SMA) and fibronectin expression by Western analysis and/or immunoelectron microscopy, ultrastructural properties by transmission electron microscopy and functional properties by collagen gel contraction and invasion assays.

**Results:**

Both pirfenidone and nintedanib reduced in vitro proliferation of fibroblastic cells in a dose dependent manner. The number of cells from control lung was reduced to 47 % (*p* = 0.04) and of IPF cells to 42 % (*p* = 0.04) by 1 mM pirfenidone and correspondingly to 67 % (*p* = 0.04) and 68 % (*p* = 0.04), by 1 μM nintedanib. If both drugs were used together, a further reduced proliferation was observed. Both pirfenidone and nintedanib were able to reduce the amount of α-SMA and the myofibroblastic appearance although the level of reduction was cell line dependent. In functional assays, the effect of both drugs was also variable.

**Conclusions:**

We conclude that the ultrastructure and function of fibroblasts and myofibroblasts are affected by pirfenidone and nintedanib. Combination of the drugs reduced cell proliferation more than either of them individually. Human lung derived cell culture systems represent a potential platform for screening and testing drugs for fibrotic diseases.

**Electronic supplementary material:**

The online version of this article (doi:10.1186/s12931-016-0328-5) contains supplementary material, which is available to authorized users.

## Background

Idiopathic pulmonary fibrosis (IPF) is a severe type of lung fibrosis with a median survival of 2–3 years [1]. The pathogenesis of IPF is still unclear, although marked progress has been made recently both in clarifying disease mechanisms and in developing new therapeutic agents. At present, no pharmacological therapy is able to cure the disease but two drugs, pirfenidone and nintedanib i.e. BIBF1120, have been shown to slow the progression of the disease [2–4] whereas the previously used N-acetylcysteine had no effect on the outcome [5, 6].

Changes in epithelial and mesenchymal cells as well as the interaction between these cells are the main characteristics of IPF whereas it is currently believed that inflammatory processes play only a minor role. One widely accepted hypothesis to explain the mechanisms in IPF pathogenesis postulates that an injury of the alveolar epithelium results in excessive production of extracellular matrix (ECM) proteins, growth and transcription factors and cytokines by fibroblasts [7]. The fibroblast focus, a typical histological feature of IPF, is a specific aggregate of cells, especially fibroblasts and myofibroblasts covered by injured and hyperplastic epithelium, and ECM produced by myofibroblasts [8]. Studies have revealed that IPF patients with a high number of fibroblast foci have a shortened survival [9]. In addition, the extent of expression of alpha smooth muscle actin (α-SMA), as a marker of myofibroblasts, in the lungs of IPF-patients, has been shown to be negatively associated with patient survival [10].

In our previous studies, we have observed that it is possible to isolate and culture fibroblast and myofibroblast containing cell lines both from the bronchoalveolar lavage (BAL) fluid and lung tissue samples of patients with different types of lung diseases including IPF. Furthermore, we characterized these cells by a variety of methods including electron and immunoelectron microscopy [11, 12]. We have noted that myofibroblasts from different lung diseases display different ultrastructural and functional properties [11, 12]. In particular, we and other investigators have observed that fibroblasts and myofibroblasts containing cells lines cultured from IPF patients are more invasive than the cells obtained from other lung diseases [11, 13]. It has also been reported that fibroblastic cells from IPF patients have a higher amount of α-SMA, a lower growth rate and a higher number of apoptotic cells than found in controls [14].

Most of the previous preclinical studies investigating the effect of potential anti-fibrosis drugs have been conducted by using animal models [15]. For example, bleomycin-induced fibrosis in mice, rats or hamsters has been the most commonly used study protocol. Although testing in animal models is rational, it is often difficult to extrapolate the results to the human diseases. For instance, bleomycin-induced fibrosis in rodents resembles rather poorly the IPF in humans [16, 17], and further, the pulmonary anatomy and cellular components of rodents and other experimental animals are very different from their human counterparts. There are very few studies which have utilized human lung cells to investigate novel therapeutic agents for pulmonary fibrosis, and even fewer that have used cells originating from IPF patients. Moreover, most of the previous studies have focused on only one pharmacological agent and not compared two or more drugs using the same study protocol. Surprisingly, the mechanisms of action of many promising anti-fibrosis drugs are still not fully understood. A better understanding of mechanism could help us selecting the most suitable therapy for each individual patient as well as in developing improved combination treatment modalities in the future [18].

The aim of the present study was to evaluate the effects of pirfenidone and nintedanib on the ultrastructural and functional properties of stromal cells such as fibroblasts and myofibroblasts collected from BAL and lung tissue samples both from patients with IPF and from control lung.

## Methods

### Study subjects

The study material comprised lung tissue from 7 patients with IPF and from 4 control patients having normal peripheral lung. The patients underwent diagnostic BAL, diagnostic surgical lung biopsy or surgery for lung cancer during 2008–2012 in Oulu University Hospital (Table 1). All control patients were nonsmokers with normal lung function and normal lung histology outside the lung tumor. Pieces of lung tissues were collected from non-involved areas outside the tumor as previously described [19]. As the cells of IPF patients were derived from diagnostic samples before the year 2012, none of the study subjects was treated with pirfenidone or nintedanib before the cells were derived.

**Table 1:** Sample information

		Control	IPF
Total number		4	7
Smoking status	Non-smoker	4	3
	Ex-smoker		3
	Smoker		1
Number of samples derived from	BAL	0	4
	Biopsy	0	2
	Lobectomy for lung cancer	4	1

### Ethics, consent and permissions

The donors were informed and interviewed before the operation. Each patient provided written informed consent. The study protocol was approved by the Ethical Committee of Northern Ostrobothnia Hospital District in Oulu (64/2001, amendment 2005, 2/2008).

### Cell culture

Cell samples were collected and stromal cells were cultured as described previously [11, 12]. Briefly, an aliquot of BAL-sample or collagenase-digested lung biopsy specimen was centrifuged (300 g, 10 min) and plated at a density of approximately 40,000 cells/cm^2^ in a medium consisting of Minimun essential medium Eagle α modification (Sigma-Aldrich, Inc, St Louis, MO, USA) supplemented with 13 % heat-inactivated fetal bovine serum (PromoCell, Heidelberg, Germany), 2 mM L-glutamine, 100 U/ml penicillin, 0.1 g/l streptomycin, 2.5 mg/l amphotericin B and 10 mM HEPES (all from Sigma-Aldrich). The cells were passaged at near-confluence and used for experiments in passages 2–5. The cells were exposed to 0.1–0.5 mM pirfenidone (Santa Cruz Biotechnology) or 0.1–0.5 μM nintedanib by adding the drug into the cell culture medium with or without serum. Pilot studies were used to select drug concentrations that were low enough not to harm the cells but high enough to cause responses. The effects of the drugs were tested also in the presence of 2–5 ng/ml transforming growth factor β1 (TGFβ1) (Sigma-Alrich) in serum-free conditions.

### Proliferation

In the proliferation assay, the cells were plated on 96-well plates with 500 cells per well, 6 parallel wells for each condition. On the next day, the medium was replaced with new medium (control medium with serum, medium with 0.1–0.5 mM pirfenidone and/or 0.1–0.5 mM nintedanib with serum, medium without serum but with 5 ng/ml TGFβ1 or serum-free medium with TGFβ1 and nintedanib or pirfenidone). The number of cells was measured after 1, 3 and 7 days of drug exposure with the MTT-assay (3-(4,5-dimethylthiazol-2-yl)-2,5-diphenyltetrazolium bromide, Sigma-Adrich). The MTT reagent was added to the wells at a final concentration 0.5 g/l. The cells were allowed to reduce MTT into formazan (2 h at 37 °C) the amount of which was measured spectrophotometrically at a wavelength of 550 nm against background (650 nm) after lysing the cells in DMSO.

### Western analysis

Western analysis of α-SMA was performed as described earlier [11]. Briefly, the cells were lysed in 50 mM Tris, 0.1 % Triton X-100, 0.9 % NaCl supplemented with a protease inhibitor cocktail tablet (Roche, Mannhaim, Germany) and 20 μg aliquots of samples were loaded and run on 12 % SDS–PAGE. The proteins were transferred onto nitrocellulose membrane (Protran, Schleicer and Schuell, Bioscience, Dassel, Germany). After blocking with milk, the membranes were incubated with a 1:1000 dilution of α-SMA antibody followed by 1:1000 diluted secondary antibody (IRDye 800 conjugated anti-mouse IgG, Rockland Immunochemicals, Gilbertsville, PA, USA). Protein intensities were detected and analyzed with an Odyssey infrared imager (Li-Cor Biosciences).

### Transmission electron microscopy

All the cultured cells have been previously characterized by transmission electron microscopy (TEM) which showed that morphologically the cell populations consisted of fibroblasts and myofibroblasts [11, 12]. In order to evaluate the effect of 0.5 mM pirfenidone and 0.5 μM nintedanib on cellular ultrastructure, the cells from control and IPF patients were exposed to the drugs for 4 days in the presence of 5 ng/ml TGFβ1. Samples treated with TGFβ1 alone were used as controls. The samples were fixed and prepared for TEM as described earlier [11]. Briefly, the cells were fixed in 1 % glutaraldehyde-4 % paraformaldehyde for 10 min. The cells were detached mechanically, pelleted and further fixed for 1 h. The cells were immersed in agarose and fixed in 1 % osmium tetroxide for 0.5 h. The pellet was immersed in Epon LX112 after dehydration in acetone. Uranyl acetate and lead citrate were used for staining ultrathin sections and the cells were examined in a Tecnai G2 Spirit transmission electron microscope.

### Immunoelectron microscopy

The cultured cells from two controls and two IPF patients were exposed to 0.5 mM pirfenidone or 0.5 mM nintedanib for 4 days in the presence of 5 ng/ml TGFβ1. Samples treated with TGFβ1 alone were used as controls. The samples were prepared as described earlier [11]. The cells were fixed in 4 % paraformaldehyde-2.5 % sucrose, immersed first in 12 % gelatin and then in 2.3 M sucrose. Ultrathin sections were incubated with monoclonal anti-human α-SMA antibody (1:1000 dilution, clone 1A4, Dako, Glostrup, Denmark) or monoclonal anti-human fibronectin antibody (1:7000 dilution, clone IST-4, Sigma-Aldrich) followed by secondary antibody and protein A-gold conjugates. Sections were embedded in methylcellulose and visualized as TEM samples.

### Collagen gel contraction assay

The contraction assay was essentially performed as described earlier [20]. Briefly, a total of 300,000 cells and 0.75 mg collagen isolated from rat tail tendon were used for each ml of gel and then 550 μl gels were cast on 24-well plates for 15 min at 37 °C after which the gel was detached and 1 ml of serum-free medium with or without drugs was added to each well. The sizes of the gels were measured daily. The solvent of each drug was used as a control (vehicle).

### Invasion

In the invasion assay, the 8 μm pore-sized Transwell inserts for 96-well plates (Corning Incorporated, Lowell, MA, USA) were coated with 50 μl of 1 g/l Matrigel (BD Biosciences) and the plates were incubated at 37 °C O/N as described previously [11]. A total of 50,000 cells per well were plated on the top of Matrigel and cell culture medium was placed into the lower chamber. 0.5 mM pirfenidone or 0.5 μM nintedanib was added to the medium and the cells were allowed to invade the Matrigel in eight parallel wells. Distinct plates for the preparation of standard curves were prepared for each sample type. After 3 days, Matrigel and cells inside the insert were removed and the inserts and standard plates were incubated in a cell culture medium containing 0.5 g/l MTT-reagent for 2 h. The MTT-reagent was removed and the cells were lysed in DMSO and the absorbance was read as in the cell proliferation assay. The number of invading cells was evaluated from standard curves.

### Statistical analysis

Statistical analysis and data visualization was performed by OriginPro 9.1 or by Statistical Package for the Social Sciences. *T*-test, Mann–Whitney test or non-parametric Kruskal-Wallis test (KW test) was used and *p*-values below 0.05 were considered as statistically significant.

## Results

### Pirfenidone and nintedanib reduced the proliferation of both control lung and IPF derived fibroblastic cells

**Fig. 1:**
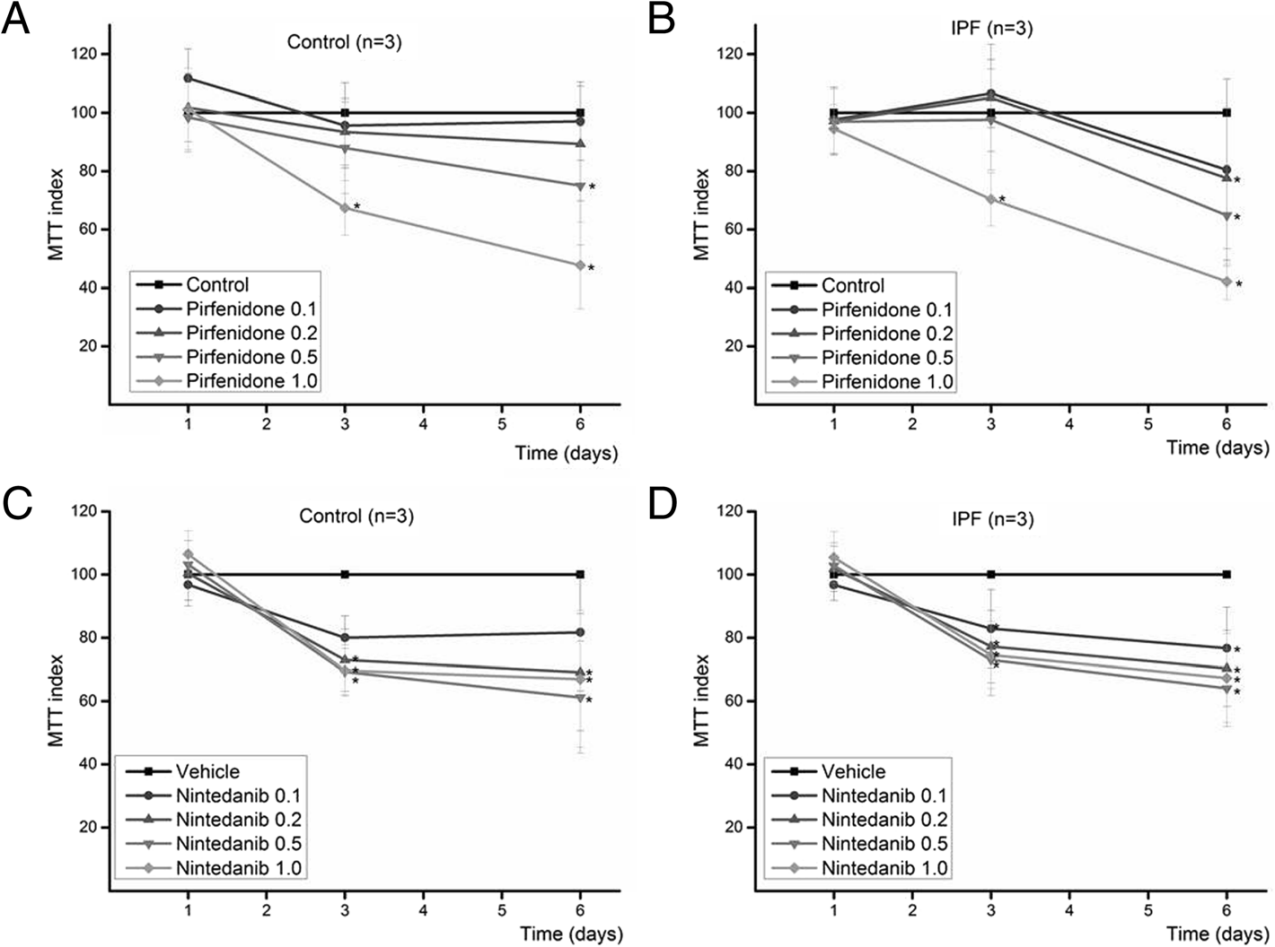
Proliferation of stromal cells was reduced by pirfenidone (**a**, **b**) and by nintedanib (**c**, **d**). Six samples were analyzed of which 3 were derived from control lung (**a**, **c**) and 3 from IPF (**b**, **d**). Different concentrations of pirfenidone (0.1 mM-1 mM) or nintedanib (0.1-1 μM) were added to the cell culture at the beginning of the experiment. The values were related to the corresponding control. (*p < 0.05, **p < 0.01, KW test)

Three stromal cell lines derived from control lung and three cell lines derived from IPF were used for proliferation assay. Both pirfenidone and nintedanib inhibited the proliferation of cells in a dose dependent manner (Fig. 1). No signs of acute toxicity were seen at the concentrations used (0.1 -1 mM pirfenidone and 0.1 -1 μM nintedanib) as can be seen from the MTT-values from day one, the drug treated samples had similar MTT indexes as control samples. On the days three and six, a significant reduction in the amount of cells could be seen, especially at higher concentrations of both drugs. On day six, the proliferation of control cells was reduced to 47 % (*p* = 0.04) and that of IPF cells to 42 % (*p* = 0.04) by 1 mM pirfenidone and to 67 % (*p* = 0.04) and 68 % (*p* = 0.04), respectively, by 1 μM nintedanib (KW test). One control sample and one IPF sample responded only to the two highest concentrations of pirfenidone whereas other four samples showed reduced proliferation already at lower doses.

As expected, when the cells were cultured in serum-free conditions in the presence of 5 ng/ml TGFβ1, the inhibitory effect of the drugs was lower, due to the slower proliferation of the cells compared to that in serum containing medium (Additional file 1). Statistically significant reductions were seen only with 0.5 mM and 1.0 mM pirfenidone. It is notable that there were rather large variations in the response especially in TGFβ1 exposed IPF derived samples, i.e. one of the samples responded to both drugs, one sample responded only to pirfenidone and one sample did not respond at all to either of the drugs. TGFβ1 exposed control cells seemed to respond to the lower concentrations of pirfenidone and nintedanib than cells from the patients with IPF but this was not statistically significant.

### Pirfenidone and nintedanib reduced the amount of α-SMA analysed by Western analysis

**Fig. 2:**
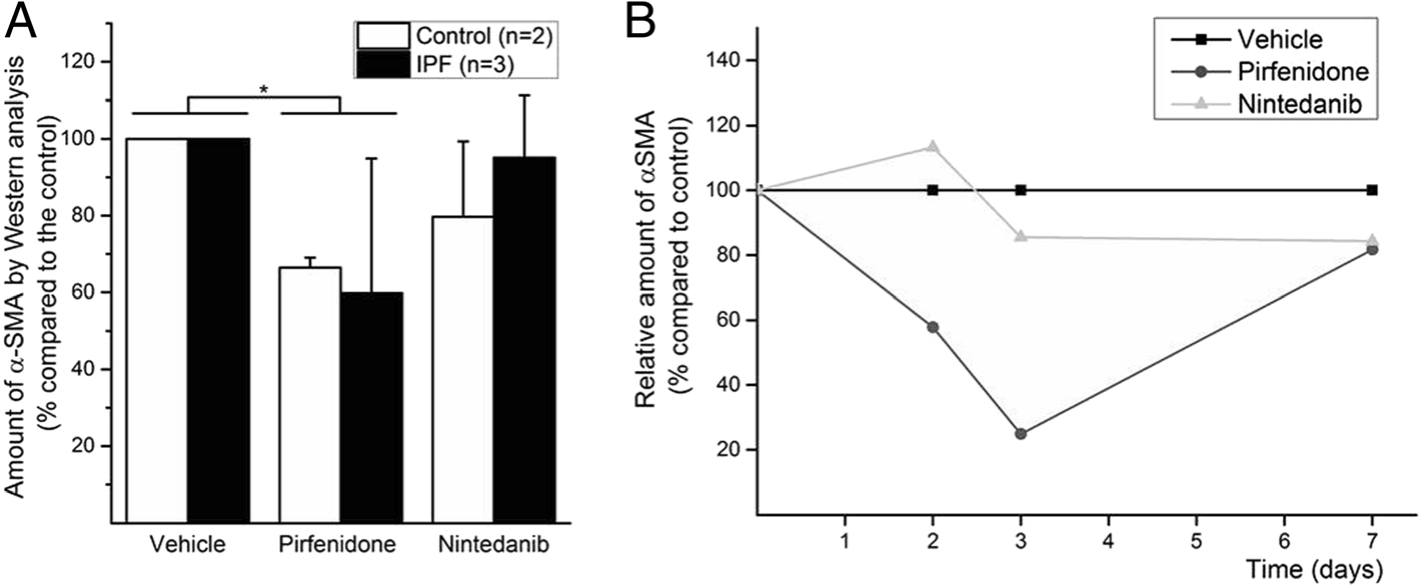
The amount of α-SMA was evaluated by Western analysis. A total of two cell lines derived from control lung and three cell lines derived from IPF lung were used for α-SMA quantification on day 3 (**a**). Pirfenidone reduced α-SMA expression significantly. One IPF derived sample was used to evaluate α-SMA expression after a single exposure to 0.5 mM pirfenidone or 0.5 μM nintedanib on days 2, 3 and 7 (**b**). (*p < 0.05, KW test)

The amount of α-SMA in cells cultured in serum-free conditions with or without drug treatment was evaluated by Western analysis (Fig. 2). Both pirfenidone and nintedanib reduced α-SMA expression, although only pirfenidone appeared to exert a statistically significant effect in these conditions at day three (control lung *n* = 2, IPF *n* = 3, fig. 2a) (*p* = 0.005). However, the α-SMA expression of IPF seemed to be restored to the control level within 1 week (Fig. 2b) if the cells were exposed to the drugs once only at the beginning of the experiment. The variation in the levels of α-SMA was high since the relative amount of α-SMA in pirfenidone treated cases compared to the corresponding sample of a control case varied from 25 to 91 % in the samples derived from different patients. In the nintedanib treated samples of IPF-patients, the corresponding amounts varied from 66 to 114 %. Pirfenidone reduced the amount of α-SMA in all cases including IPF and controls, while nintedanib reduced it in four cases (two IPF, two controls) and actually slightly increased the amount of α-SMA in one IPF-case.

### Pirfenidone and nintedanib reduced myofibroblastic ultrastructural features as visualized by transmission electron microscopy

**Fig. 3:**
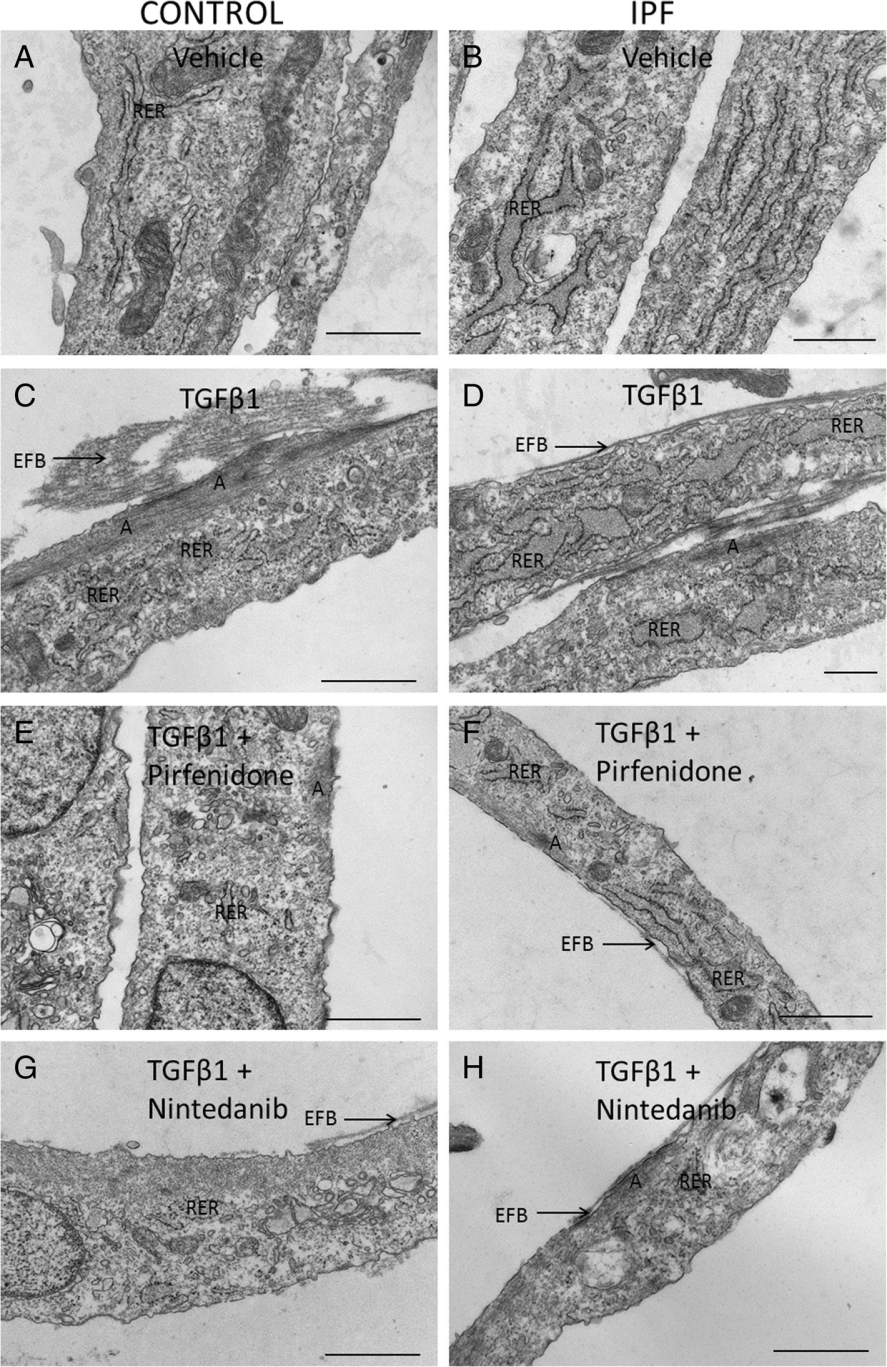
Transmission electron microscopy analysis of stromal cells derived from control lung (**a**, **c**, **e**, **g**) or IPF lung (**b**, **d**, **f**, **h**). Under normal cell culture conditions **a**, **b** there were only occasional myofibroblast features detected. TGFβ1 (5 ng/ml) **c**, **d** increased the myofibroblastic appearance such as the extracellular filament bundle (EFB), intracellular actin filament (**a**) and dilated rough endoplasmic reticulum (RER). Both 0.5 mM pirfenidone (**e**, **f**) and 0.5 μM nintedanib (**g**, **h**) reduced the numbers of TGFβ1 induced myofibroblastic features. Scale bar is 1 μm

Prior to TEM, the cells from one control and from one IPF lung were cultured without serum for 4 days with 5 ng/ml TGFβ1 alone, with TGFβ1 and 0.5 mM pirfenidone, with TGFβ1 and 0.5 μM nintedanib or without any added compounds. In the untreated samples, it was possible to detect only occasionally the typical ultrastructural features of myofibroblasts, i.e. prominent intracellular actin belt and extracellular fibronectin which compose the fibronexus (FNX) cell surface structure as well as the dilated rough endoplasmic reticulum (RER). The cell population mainly consisted of fibroblasts but occasional myofibroblast features were detected in both IPF and control lung derived cells (Fig. 3a and b). TGFβ1 induced both control and IPF lung derived cells to express more often the typical ultrastructural features of myofibroblasts (Fig. 3c and d). Both pirfenidone (Fig. 3e and f) and nintedanib (Fig. 3g and h) reduced the amount of myofibroblastic ultrastructural features. In particular, the extracellular component of fibronexus was fragile compared to the control samples treated only with TGFβ1.

### Pirfenidone and nintedanib had variable effects on the expressions of α-SMA and fibronectin analysed by immunoelectron microscopy

**Fig. 4:**
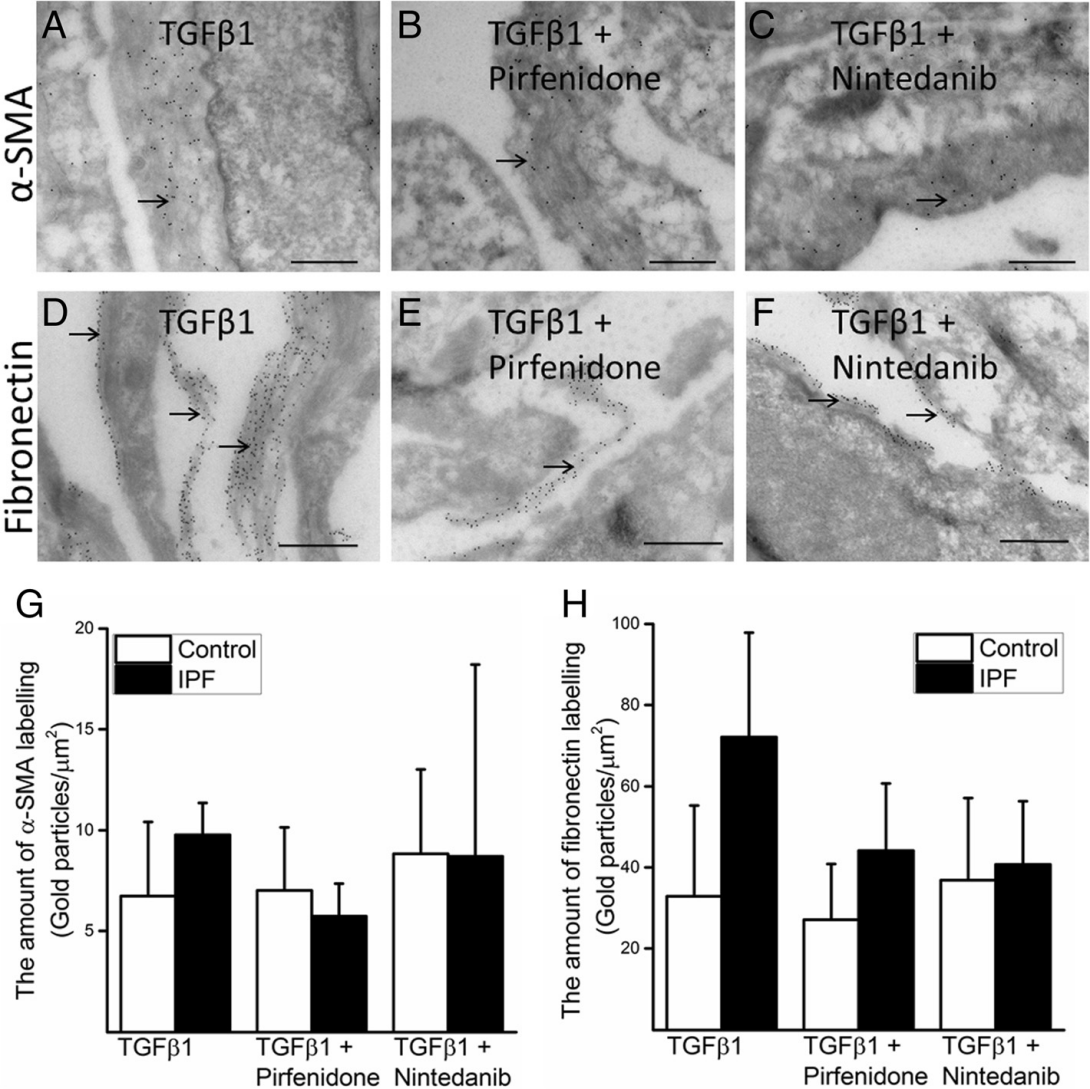
A representative of IEM of IPF derived cells (**a**-**f**) and quantification of α-SMA (**g**) and fibronectin (**h**). The cells were exposed to TGFβ1 (**a**, **d**), to TGFβ1 and pirfenidone (**b**, **e**) and to TGFβ1 and nintedanib (**c**, **f**). α-SMA (**a**-**c**) and fibronectin (**d**-**f**) expression can be localized by the presence of gold particles (black dots) by IEM. For evaluating the level of expression (**g**) and (**h**), the values of average numbers of gold particles in stromal cells derived from two control and two IPF lungs are shown. The concentrations used were 5 ng/ml TGFβ1, 0.5 mM pirfenidone and 0.5 μM nintedanib. Scale bar is 0.5 μm

The amounts of α-SMA and fibronectin in individual cells were evaluated by IEM (Fig. 4) from two control and two IPF lung derived cell lines. α-SMA positive labeling in the actin belts below the cell membrane was observed in all samples treated with 5 ng/ml TGFβ1 (Fig. 4a). In both control cell lines, the amount of α-SMA positive labeling was not significantly affected by either 0.5 mM pirfenidone or 0.5 μM nintedanib (Fig. 4g). A minor reduction in the amount of α-SMA was seen in both IPF derived cell lines exposed to pirfenidone but only in one sample treated with nintedanib (Fig. 4b, c and g). TGFβ1 exposed cells from IPF seemed to have more extracellular fibronectin labeling than the cells from control lung (Fig. 4h). Both pirfenidone and nintedanib reduced fibronectin labelling in both IPF samples while no major effect was visible on control cells (Fig. 4d, e, f and h).

### Pirfenidone and nintedanib had variable effects on collagen gel contraction

**Fig. 5:**
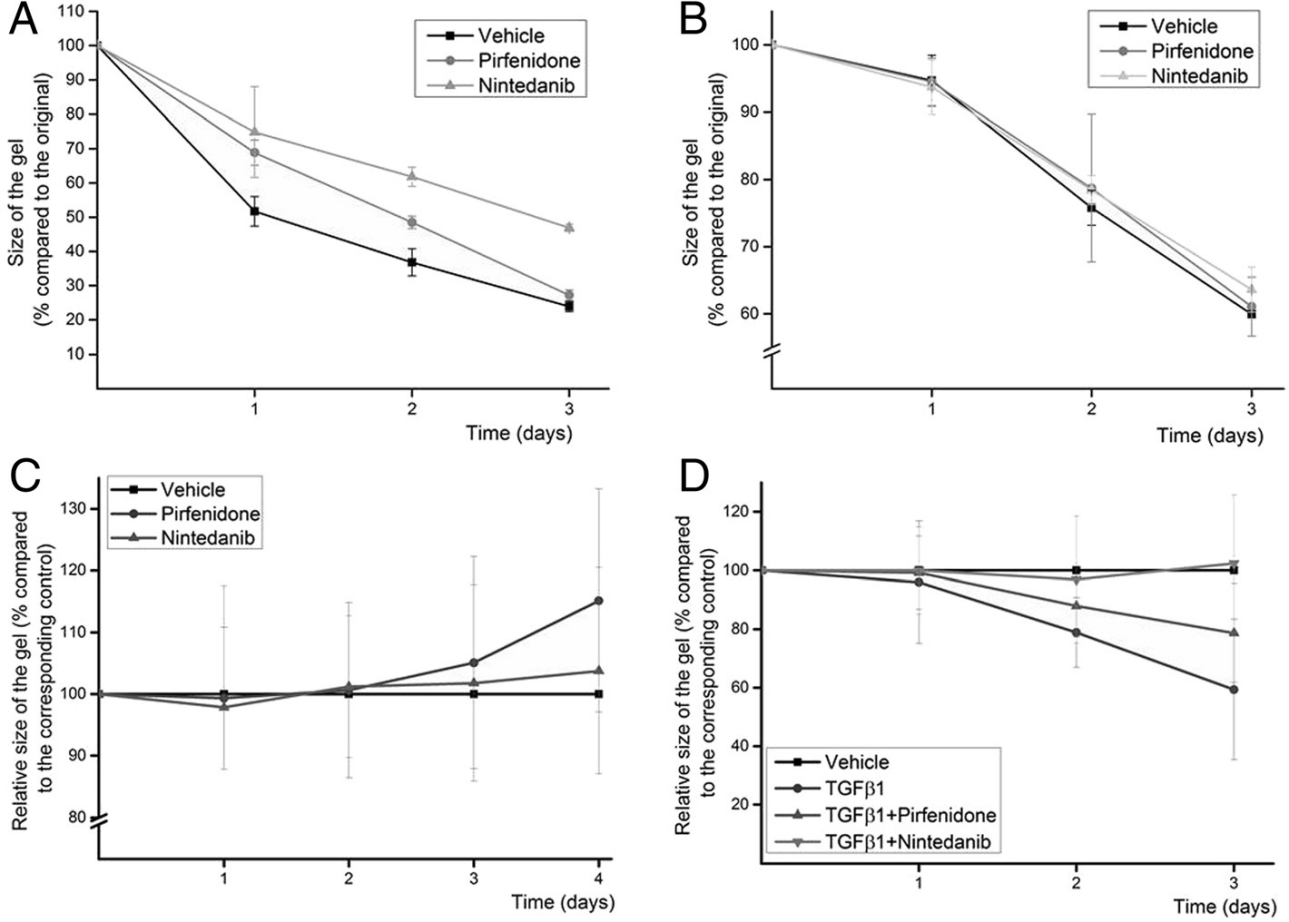
Collagen gel contraction assay of IPF derived cells from two different patients are shown (**a** and **b**). The values shown are gel sizes compared to the original size. Averages of relative gel sizes related to the corresponding control at each time point of samples derived from seven patients with IPF (**c**). Relative values from four cell lines treated with TGFβ1 alone, TGFβ1 and pirfenidone or TGFβ1 and nintedanib (**d**). The concentrations used were 5 ng/ml TGFβ1, 0.5 mM pirfenidone and 0.5 μM nintedanib

The effect of pirfenidone and nintedanib was tested in the collagen gel contraction assay (Fig. 5). The results were variable since in some IPF-cases both 0.5 mM pirfenidone and 0.5 μM nintedanib inhibited contraction (Fig. 5a) while in some other IPF-cases where the cells had a lower contraction capacity, neither of the drugs exerted any visible effect (Fig. 5b). On average in the samples of seven IPF-patients, it was apparent that especially pirfenidone reduced the extent of contraction, although this was not statistically significant (Fig. 5c). However, a more prominent inhibition of contraction was seen by both pirfenidone and nintedanib if the cells were exposed to 5 ng/ml TGFβ1 (*n* = 4) (Fig. 5d). In general, pirfenidone inhibited contraction more than nintedanib with or without the presence of TGFβ1 but due to variation between different samples, these differences are not statistically significant.

As the amount of cells affects the results, the effect of the drugs on cell numbers was analyzed by the MTT assay under gel contraction conditions, i.e. without serum. Under these conditions, the tested drugs did not exert any effect on the amounts of cells at the concentrations used which were selected to be non-toxic (Additional file 2).

### Invasion was slightly affected by pirfenidone and nintedanib

**Fig. 6:**
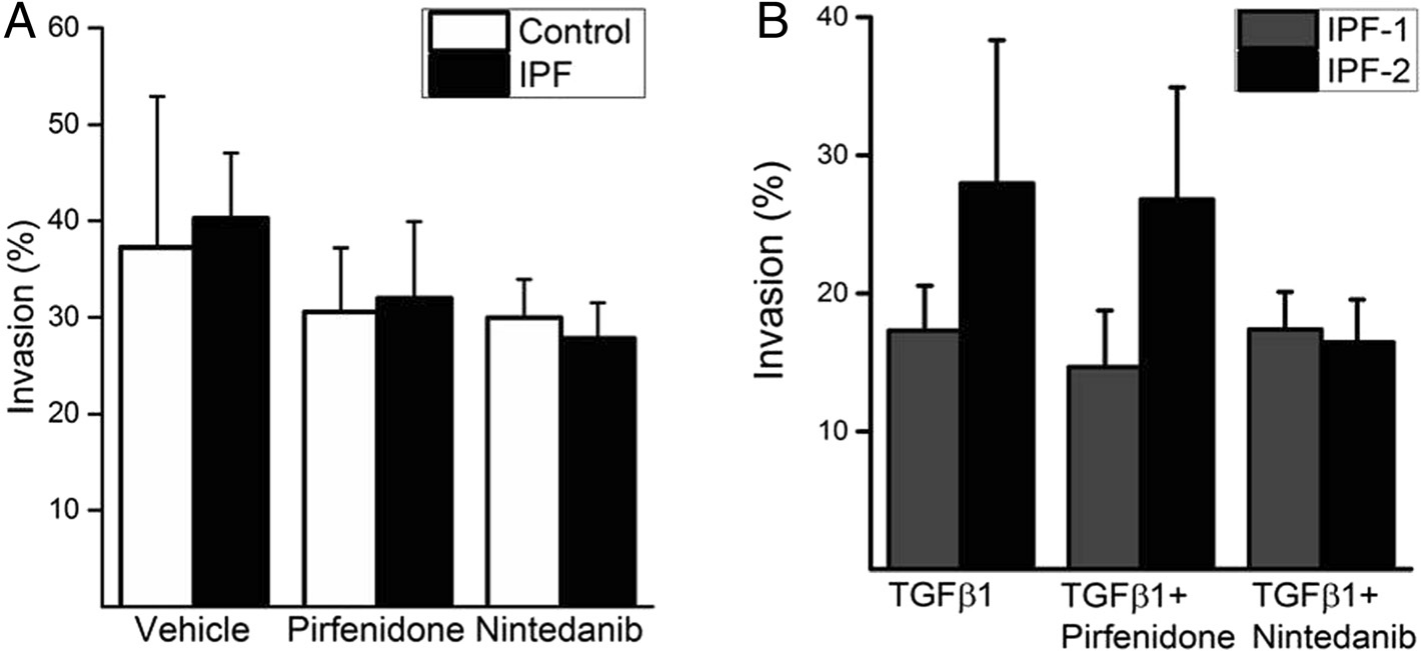
Invasion assay. Invasion percentages of both healthy control lung (white bar) and IPF lung (black bar) are shown with or without pirfenidone or nintedanib (**a**). Two IPF lung derived samples were exposed to TGFβ1, to TGFβ1 and pirfenidone, or to TGFβ1 and nintedanib (**b**)

One cell line from control lung and one from IPF lung were tested in the Matrigel invasion assay. Cells were allowed to invade in a medium containing serum in the presence or absence of the drugs (Fig. 6a). Both 0.5 mM pirfenidone and 0.5 μM nintedanib seemed to reduce invasion (but this difference was not statistically significant) and this effect was more pronounced in IPF cells than in controls.

In addition, two samples from IPF lung were analyzed under serum free conditions with TGFβ1 (Fig. 6b). Nintedanib reduced the invasion capabilities of one sample but the other sample showed similar properties with the drugs as without their presence. Pirfenidone did not markedly reduce invasion in either of the cases.

### Combination of nintedanib and pirfenidone reduces proliferation more than individual drugs

**Fig. 7:**
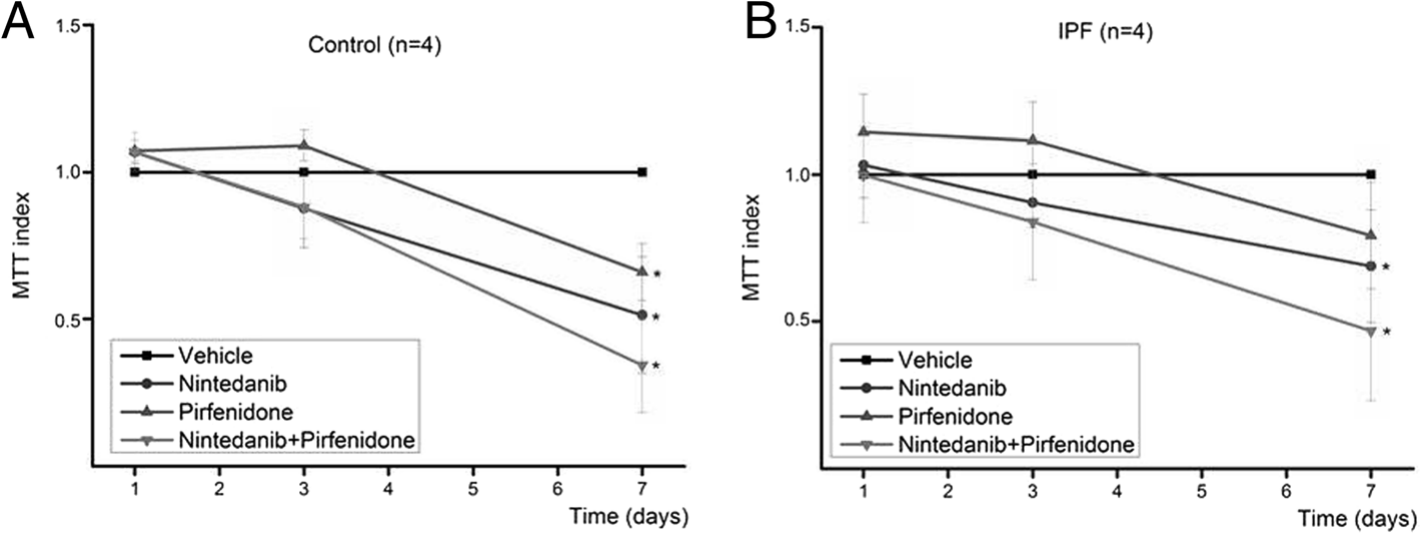
Effect of combined therapy on cell proliferation. The stromal cells from four control lungs (**a**) and four IPF lungs (**b**) were treated with 0.5 mM pirfenidone, 0.5 μM nintedanib or both drugs simultaneously. (*p < 0.05, KW test)

As both pirfenidone and nintedanib significantly reduced the proliferation, we also tested their effect on proliferation when both drugs were used at the same time for the cell lines derived from IPF (*n* = 4) lung and control lung (*n* = 4). The cells were treated with 0.5 mM pirfenidone, with 0.5 μM nintedanib or with combination of both drugs. In both control lung and IPF derived cells, the proliferation was slowest if both drugs were used (Fig. 7). As previously, the drugs were administered only once at the beginning of the study but the effect on proliferation was most prominent at day 7. On day seven, the proliferation of control cells was reduced to 66 % (*p* = 0.01) and that of IPF cells to 79 % (*p* = 0.01) by pirfenidone and to 51 % (*p* = 0.01) and 69 %, respectively, by nintedanib. The combination of both drugs reduced the proliferation to 34 % (*p* = 0.01) in control cells and to 47 % (*p* = 0.01) in IPF derived cells.

## Discussion

Our study demonstrates that the effects of both pirfenidone and nintedanib can be evaluated on cultured cells derived from control or IPF lung. These drugs affected not only the proliferation rate of the cells but they also inhibited myofibroblastic ultrastructural features, affected contraction of three-dimensional collagen gels and the invasive capabilities of the cells. Originally myofibroblasts were discovered during EM investigations of a healing wound [21]. Although α-SMA is the most common marker for myofibroblasts, it is not specific since other types of cells, such as smooth muscle cells, are known to express α-SMA. So far, there is no specific marker available for the myofibroblast, and thus an EM assessment is still needed for the ultimate identification of this cell population. A typical ultrastructural feature for the myofibroblast is a fibronexus (FNX) which is composed of intracellular α-SMA and the associated extracellular fibronectin [22]. As far as we are aware, this is the first study in which myofibroblasts cultured from IPF patients have been examined by TEM and IEM focusing on FNX in an experimental induction model which has included exposure to anti-fibrosis drugs.

Many of previous studies on pirfenidone or nintedanib have been conducted using the bleomycin-induced fibrosis model in experimental animals, like mice and hamsters. These have revealed that pirfenidone decreased the hydroxyproline level as well as reduced the extent of fibrosis and the numbers of myofibroblasts in lung [23, 24]. Nakayama and co-workers used commercial human fibroblasts and noted that pirfenidone diminished the expression of heat shock protein 47 (HSP47) and collagen I after TGFβ treatment [25]. Conte and co-workers reported that primary fibroblasts collected from the human lung responded to pirfenidone in vitro [26]. Pirfenidone has been found to reduce fibroblast proliferation, TGFβ induced α-SMA and procollagen-I mRNA and protein levels, and it also inhibited the expression of factors in the TGFβ pathway [26, 27]. Similarly, it has been shown that pirfenidone inhibited collagen gel contraction and TGFβ1 induced α-SMA production of keloid derived fibroblasts [28]. The results of all of the above studies are consistent with and supported by the present data showing that pirfenidone decreased contraction capabilities, invasion and proliferation of the cells as well as the amount of α-SMA assessed by Western analysis in cell lines composed of fibroblasts and myofibroblasts. Furthermore, our findings were confirmed by an examination of individual myofibroblasts by IEM and TEM, in which the typical ultrastructural features of myofibroblasts were diminished after exposure to either nintedanib or pirfenidone.

In the present study, both nintedanib and pirfenidone inhibited TGFβ1 induced myofibroblast transformation. Previously it has been shown that nintedanib can induce simultaneous inhibition of several targets, i.e. platelet derived growth factor (PDGF), vascular endothelial growth factor (VEGF) and fibroblast growth factor (FGF). Two previous studies examining nintedanib have used the bleomycin-induced fibrosis model of rats and fibroblastic cell lines cultured from the patients with IPF, sarcoidosis and normal lung [29] and bleomycin- and silica-induced fibrosis models of mice and normal human lung fibroblasts [30]. Chaudhary and co-authors administered BIBF1000, a compound that resembles nintedanib, and demonstrated that the drug decreased the levels of fibrosis, TGFβ1, procollagen type I, fibronectin and connective tissue growth factor (CTGF) in lung tissue. In addition, they investigated the effect of BIBF1000 on primary lung fibroblasts revealing that the therapy decreased amount of α-SMA when this was estimated by Western analysis which they interpreted as a reduction in the numbers of fibroblasts differentiating into myofibroblasts [29]. The study of Wollin and others showed that nintedanib inhibited the PDGF-induced phosphorylations of PDGF-receptors α and β as well as the proliferation of human lung fibroblasts. The α-SMA gene expression was decreased in TGFβ induced fibroblasts. In lung tissue of mice, nintedanib reduced total lung collagen levels, as well as the extent of inflammation and fibrosis and prevented granuloma formation [30]. A recent study suggested that nintedanib can activate autophagic pathways in fibroblasts [31]. The results of those studies are in agreement with ours which showed that nintedanib inhibited TGFβ induced myofibroblast transformation, contraction and invasion of cells and that both pirfenidone and nintedanib could reduce the ultrastructural features of individual myofibroblasts by TEM and IEM.

Our results suggest that combination of pirfenidone and nintedanib might provide enhanced efficacy in suppressing the proliferation of fibroblastic cells. However, there are no clinical studies supporting this result. Recent pilot study evaluated safety and pharmacokinetics of combined therapy [32] and another pilot study evaluated switching the therapy from pirfenidone to nintedanib [33]. Both of these studied contained only few patients receiving both drugs and further evaluations are required, especially as the adverse effect of these drugs partially overlap.

Clinical trials and practical experience with both agents investigated in the present study have shown that the effect of treatment on each patient is variable i.e. some patients benefit more than others from either pirfenidone or nintedanib [34]. Currently, there are no biomarkers that can predict which patients will benefit from a particular therapeutic option. Interestingly, we noted in our study that also with these in vitro models, the results were variable when using cells cultured from several different IPF-patients. It is not known whether this in vitro phenomenon reflects the clinical behavior of the disease in certain IPF patients. In order to test this tempting hypothesis it would be necessary to prospectively recruit the IPF-patients prior to initiation of medication with these particular drugs. In vitro experimental exposures should be conducted in parallel with the therapeutic treatment of the patients and then the in vitro results could be compared with the clinical follow-up data. In the future, it may be possible that bronchoalveolar lavage samples could be collected from IPF-patients to determine whether they are likely to benefit from some particular type of therapy.

Pirfenidone and nintedanib reduced proliferation, the amount of α-SMA, collagen gel contraction properties, invasion capabilities and myofibroblastic-like ultrastructural features of cultured stromal cells derived from either healthy lung or from the lungs of patients with IPF. Proliferation was even further reduced if the cells were treated with both rugs simultaneously. Minor differences were observed between the cells obtained from control lung or from IPF as well as in the effects exerted by either pirfenidone or nintedanib. It is possible that some of these differences are caused by variable cell populations as it is known that IPF derived cells have more myofibroblast features than cells derived from normal lung [11]. Cell culture based in vitro platforms may be useful for screening new IPF drugs in the future. The effects of the therapeutic agents were, however, variable in the samples collected from different individuals, and therefore further studies will be needed to clarify these phenomena and to determine whether the behavior of cells in vitro accurately reflects the clinical effectiveness of a pharmacological treatment.

## Conclusions

Combination of pirfenidone and nintedanib reduced in vitro proliferation of stromal cells more than pirfenidone or nintedanib alone. Both drugs affected collagen gel contraction potency, invasion capacity and myofibroblast features of cultured stromal cells derived from either healthy or IPF lung. We believe that cell culture based in vitro platforms could be used for screening new IPF drugs in the future. Effects of the drugs are, however, variable in different samples and therefore further studies are required for understanding this phenomenon.

## Additional files

Additional file 1:
**Proliferation of TGFβ1 induced stromal cells by pirfenidone (A, B) and by nintedanib (C, D).** Six samples were analysed and 3 of them were derived from healthy lung (A, C) and 3 from IPF (B, D). Pirfenidone (0.1 mM-1 mM) or nintedanib (0.1-1 μM) was added to the sample together with 5 ng/ml TGFβ1 at the beginning of the experiment. The values were related to the corresponding control. (PDF 257 kb) Additional file 2:
**Proliferation assay performed in collagen gel contraction assay conditions.** In these conditions the cells are not proliferating. Serum was used as a positive control to induce the proliferation. (PDF 178 kb)

## Abbreviations

α-SMA: Alpha smooth muscle actin
BAL: Bronchoalveolar lavage
MTT: 3-(4,5-dimethylthiazol-2-yl)-2,5-diphenyltetrazolium bromide
ECM: Extracellular matrix
FGF: Fibroblast growth factor
FNX: Fibronexus
IPF: Idiopathic pulmonary fibrosis
IEM: Immunoelectron microscopy
KW test: Kruskal-Wallis test
PDGF: Platelet derived growth factor
TGFβ1: Transforming growth factor beta 1
TEM: Transmission electron microscopy
RER: Rough endoplasmic reticulum
VEGF: Vascular endothelial growth factor.
