# Supplementary material for: Pirfenidone and nintedanib modulate properties of fibroblasts and myofibroblasts in idiopathic pulmonary fibrosis
Source: Respir Res. 2016 Feb 4;17:14. doi: 10.1186/s12931-016-0328-5 (PMC4743320; doi:10.1186/s12931-016-0328-5)
Supplement: Additional file 1: — Proliferation of TGFβ1 induced stromal cells by pirfenidone (A, B) and by nintedanib (C, D). Six samples were analysed and 3 of them were derived from healthy lung (A, C) and 3 from IPF (B, D). Pirfenidone (0.1 mM-1 mM) or nintedanib (0.1-1 μM) was added to the sample together with 5 ng/ml TGFβ1 at the beginning of the experiment. The values were related to the corresponding control. (PDF 257 kb) [file 12931_2016_328_MOESM1_ESM.pdf]

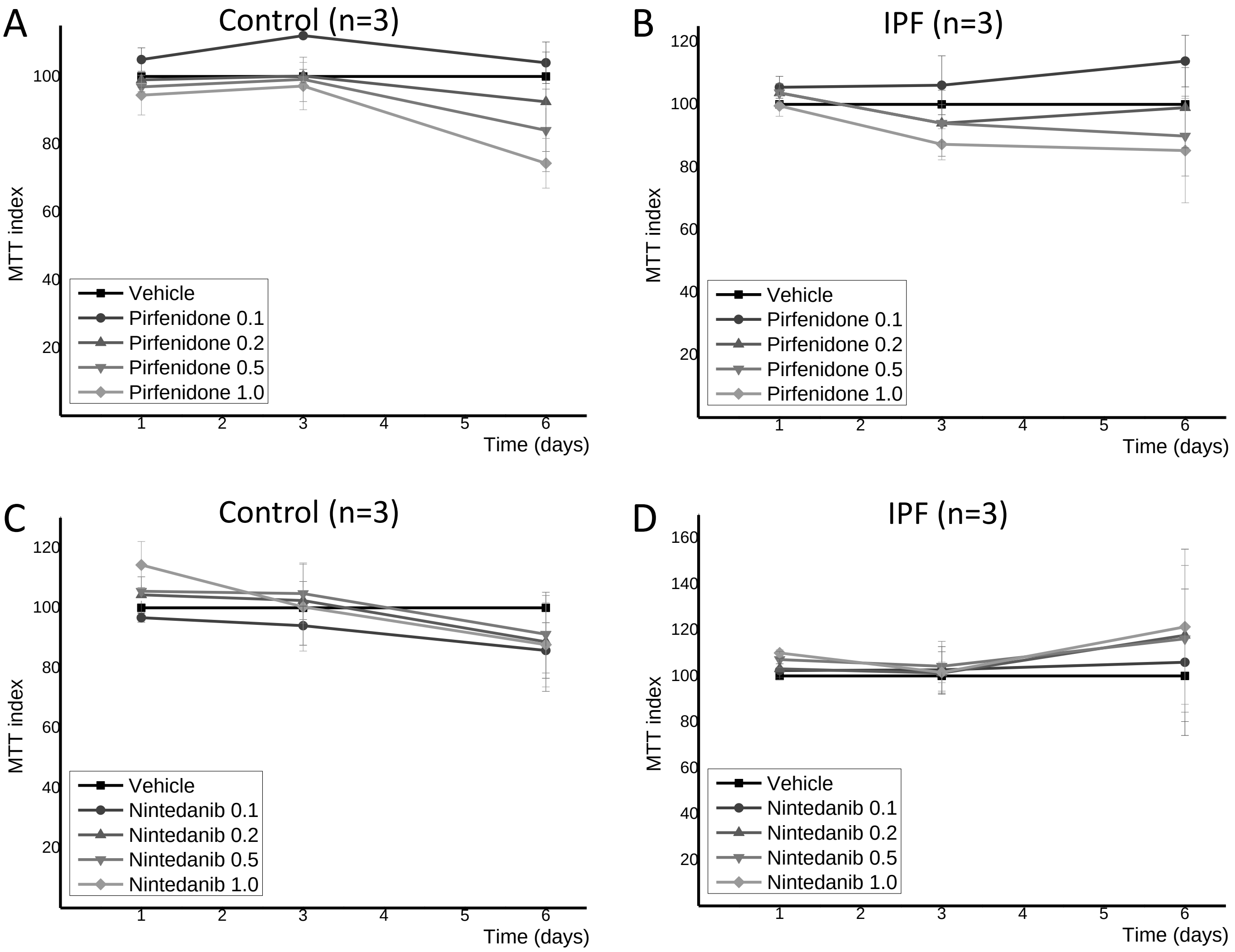

Additional file 1. Proliferation of TGFβ1 induced stromal cells by pirfenidone (A, B) and by nintedanib (C, D). Six samples were analysed and 3 of them were derived from healthy lung (A, C) and 3 from IPF (B, D). Pirfenidone (0.1 mM-1mM) or nintedanib (0.1-1 µM) was added to the sample together with 5 ng/ml TGFβ1 at the beginning of the experiment. The values were related to the corresponding control.
