# Supplementary material for: Pirfenidone and nintedanib modulate properties of fibroblasts and myofibroblasts in idiopathic pulmonary fibrosis
Source: Respir Res. 2016 Feb 4;17:14. doi: 10.1186/s12931-016-0328-5 (PMC4743320; doi:10.1186/s12931-016-0328-5)
Supplement: Additional file 2: — Proliferation assay performed in collagen gel contraction assay conditions. In these conditions the cells are not proliferating. Serum was used as a positive control to induce the proliferation. (PDF 178 kb) [file 12931_2016_328_MOESM2_ESM.pdf]

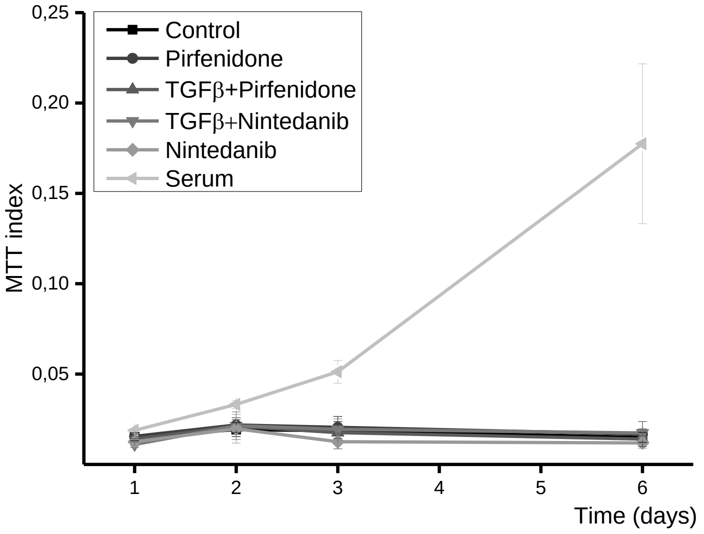

Additional file 2. Proliferation assay performed in collagen gel contraction assay conditions. In these conditions the cells are not proliferating. Serum was used as a positive control to induce the proliferation.
